# Supplementary material for: The Roles of transformer-2 (tra-2) in the Sex Determination and Fertility of Riptortus pedestris, a Hemimetabolous Agricultural Pest
Source: Insects. 2023 Oct 24;14(11):834. doi: 10.3390/insects14110834 (PMC10672195; doi:10.3390/insects14110834)
Supplement: Supplementary file 1 [file insects-14-00834-s001.zip › insects-2582903-supplementary.docx]

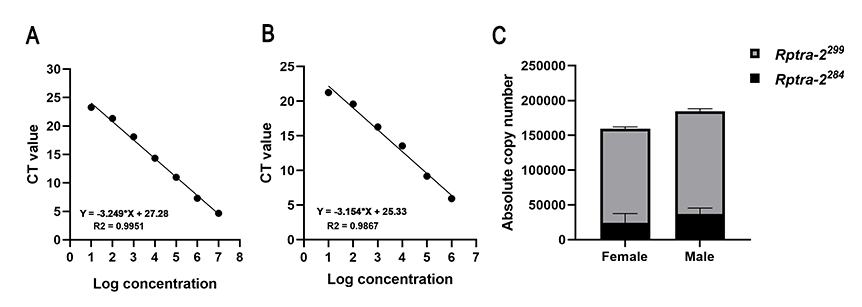


**Figure S1.** Expression of *Rptra-2*. (A) Standard curves for *Rptra-2^284^* specific primers. (B) Standard curves for *Rptra-2^299^* specific primers. (C) Expression of *Rptra-2^284^*(black) and *Rptra-2^299^* (white) in female and male insects.


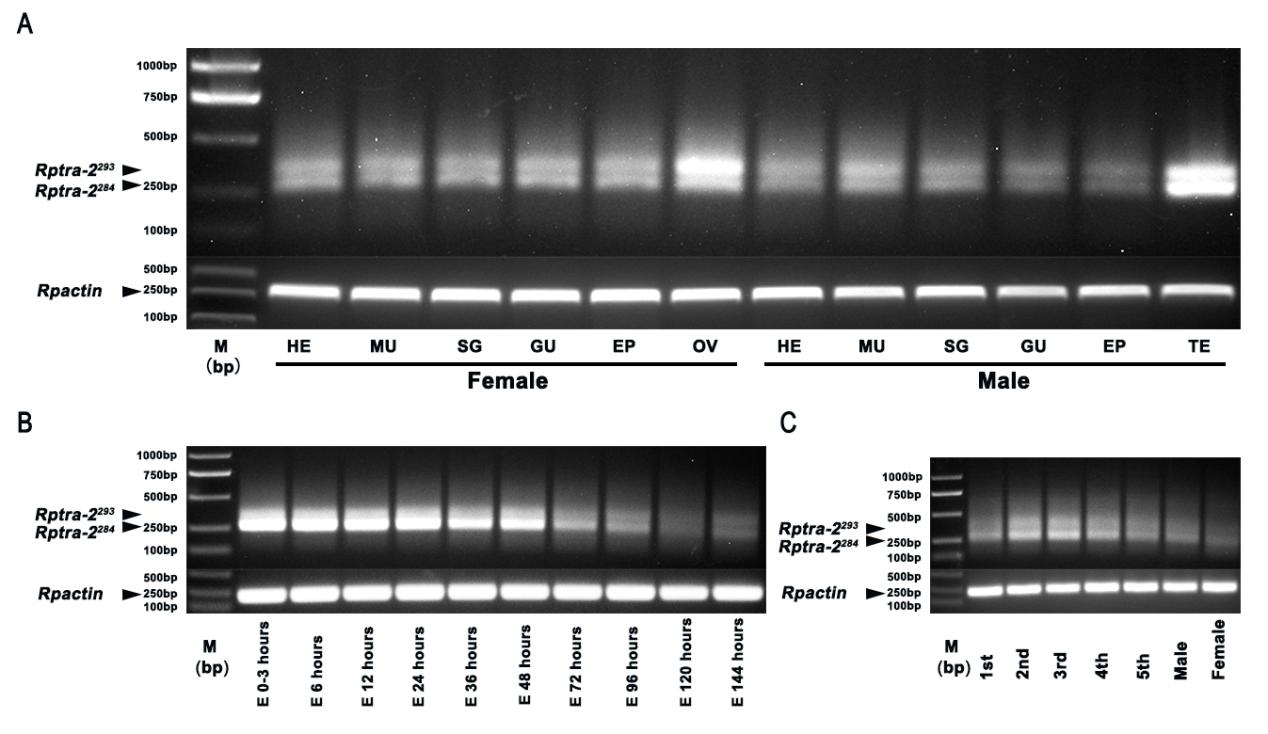


**Figure S2.** Expression of *Rptra-2^284^* and *Rptra-2^293^* in different developmental stages and tissues. (a) Detection of transcripts of *Rptra-2^284^* and *Rptra-2^293^* at different tissues of female and male (HE, head; MU, muscle; SG, salivary glands; GU, gut; EP, epidermis; OV, ovary; TE, testis). (b) Detect the transcripts of *Rptra-2^284^* and *Rptra-2^293^* at different developmental stages (E, embryonic). (c) Detect the transcripts of *Rptra-2^284^* and *Rptra-2^293^* in the following different stages: first, second, third, fourth, fifth-instar nymphs, female and male adults. Gene *Rpactin* was used as a positive PCR control.

**
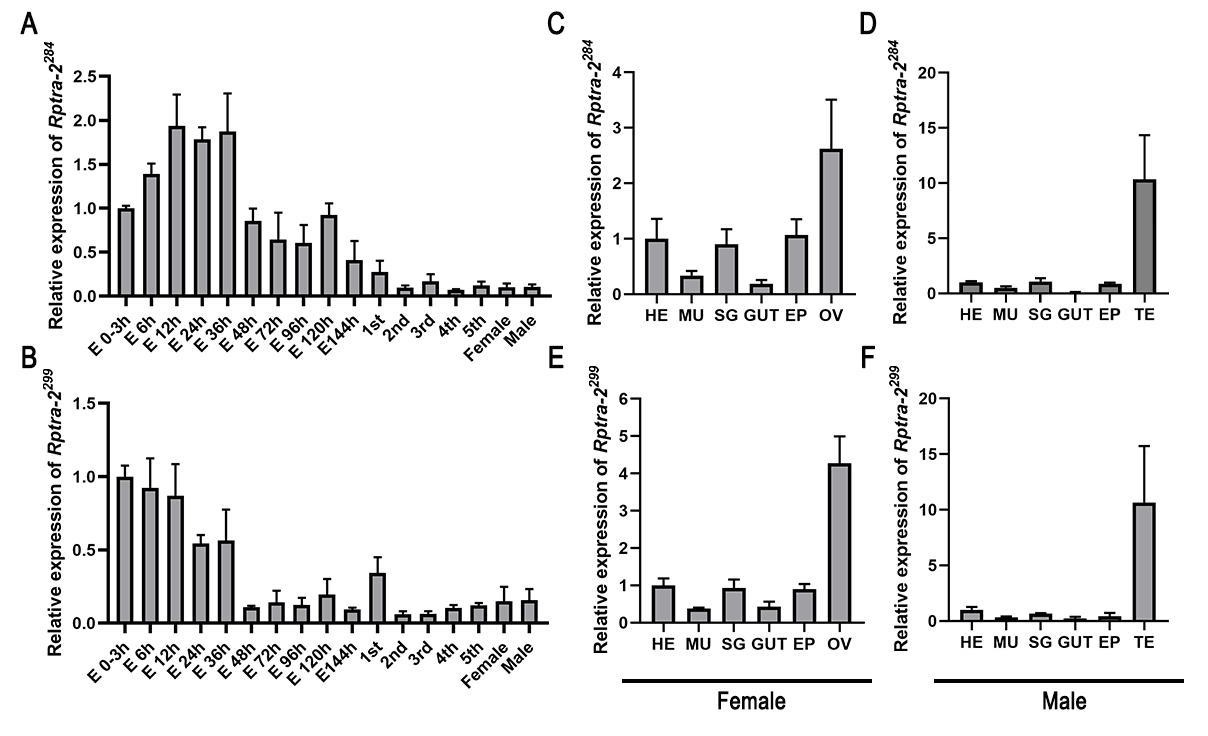
**

**Figure S3.** Expression of *Rptra-2^284^* and *Rptra-2^299^* in different developmental stages and tissues.

(A-B) Detect the relative expression of *Rptra-2^284^* (A) and *Rptra-2^299^* (B) at different developmental stages, including eggs (n = 20), different larval instars: first (n = 8), second (n = 6), third (n = 5), fourth (n = 3), fifth instar (n = 3), male adults (n = 3), and female adults (n = 3) by qRT-PCR. (C-F) Detection the relative expression of *Rptra-2^284^* (C, D) and *Rptra-2^299^* (E, F) at different tissues of female and male (HE, head; MU, muscle; SG, salivary glands; GU, gut; EP, epidermis; OV, ovary; TE, testis). The *Rpactin* gene was used as a reference gene to normalize *Rptra-2^284^* and *Rptra-2^299^* gene expression level. All treatments were independently replicated three times.


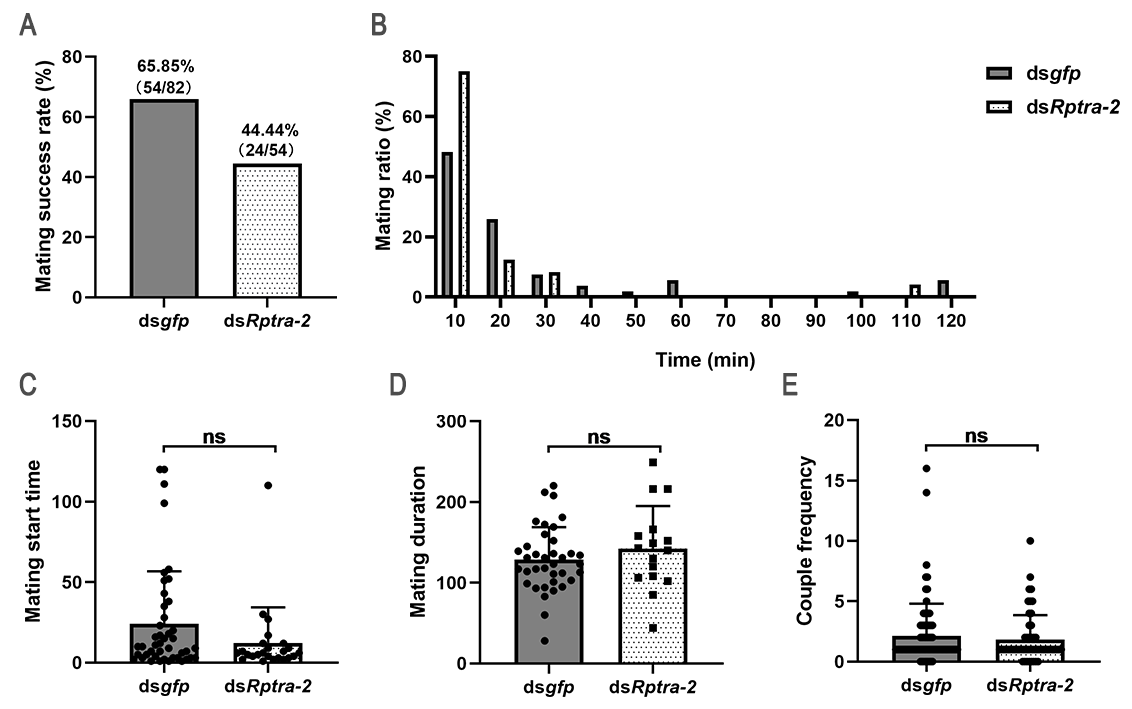


**Figure S4.** Effect of *Rptra-2* knockout on mating of *R. pedestris*. (A) Mating success ratio of ds*gfp* (n = 82)/ds*Rptra-2* males (n = 54) mating with virgin females. (B) Mating ratio of ds*gfp* (n = 44)/ds*Rptra-2* males (n = 24) mating with virgin females. (C) Mating start time of ds*gfp* (n = 44)/ds*Rptra-2* males (n = 24) mating with virgin females. (D) Mating duration of ds*gfp* (n = 37)/ds*Rptra-2* males (n = 16) mating with virgin females. (E) Couple frequency of ds*gfp* (n = 82)/ds*Rptra-2* males (n = 54) mating with virgin females. Student’s t-test was used (ns indicates no significant difference).

| **Primer name** | Primer sequence |
| --- | --- |
| **CDS-*Rptra2*-S** | TAGTGTTGCTTTGTTTTCAGGTTCC |
| **CDS-*Rptra2*-AS** | AATGTATAACTCCTTAGGCGTTTGA |
| **Rtpcr-*Rptra2*-S** | ATACAGACGGTACTCTCGCTCT |
| **Rtpcr-*Rptra2*-AS** | AACGACTTGAACACTCTCAACT |
| **qRtpcr-*Rptra2*-S** | AGACAGAAGCGAAGGAGAAAG |
| **qRtpcr-*Rptra2*-AS** | AGGCGAACGTGACCTTGAACGA |
| **qRtpcr-*Rpactin*-S** | CTCCTGAGTCAAGCACAATA |
| **qRtpcr-*Rpactin*-AS** | GCATCACACCTTCTACAATG |
| **Rtpcr*-Rptra2*-293-S** | ACAGAAAAATGAAGAAGT |
| **Rtpcr*-Rptra2*-293-AS** | ATATACGAAACAGAATCC |
| **ds*Rptra2*-S** | TAATACGACTCACTATAGGGAGAGACAACCCAGAACCCAATC |
| **ds*Rptra2*-AS** | TAATACGACTCACTATAGGGAGACCACCGTAGTAATCCCCAT |
| **ds*gfp*-S** | TAATACGACTCACTATAGGGAGAACGTAAACGGCCACAAGTTC |
| **ds*gfp*-AS** | TAATACGACTCACTATAGGGAGATGTTCTGCTGGTAGTGGTCG |
| **ds*Rptra2*-S-2** | TAATACGACTCACTATAGGGAGAGAAGATGAGTGACCGGGAGG |
| **ds*Rptra2*-AS-2** | TAATACGACTCACTATAGGGAGACTGTTACCATTGTGCCGACG |
| **qRtpcr-*Rptra-2^284^*-S** | CAATGGTAACAGAGACAACCC |
| **qRtpcr-*Rptra-2^284^*-AS** | CAGAATCCTCTTGAGTAACCAG |
| **qRtpcr-*Rptra-2^299^*-S** | CGAGATAGAGACTATGGGGA |
| **qRtpcr-*Rptra-2^299^*-AS** | TCATCCATTCCTTCTTCGTAA |

**Table S1 The primer sequences used in this article**
